# Left and right ventricular strain using fast strain-encoded cardiovascular magnetic resonance for the diagnostic classification of patients with chronic non-ischemic heart failure due to dilated, hypertrophic cardiomyopathy or cardiac amyloidosis

**DOI:** 10.1186/s12968-021-00711-w

**Published:** 2021-04-05

**Authors:** Henning Steen, Sorin Giusca, Moritz Montenbruck, Amit R. Patel, Burkert Pieske, Andre Florian, Jennifer Erley, Sebastian Kelle, Grigorios Korosoglou

**Affiliations:** 1 Department of Cardiology, Marien Hospital Hamburg, Hamburg, Germany; 2 Departments of Cardiology, Vascular Medicine and Pneumology, GRN Academic Teaching Hospital Weinheim, Roentgenstrasse 1, 69469 Weinheim, Germany; 3 Department of Medicine, University of Chicago, Illinois, USA; 4 Department of Internal Medicine/Cardiology, German Heart Center Berlin, Berlin, Germany; 5 DZHK (German Center for Cardiovascular Research), Partner Site Berlin, Berlin, Germany; 6 Departments of Cardiology, Angiology and Pneumology, Heidelberg University, Berlin, Germany; 7 DZHK (German Center for Cardiovascular Research), Partner Site Heidelberg/Mannheim, Berlin, Germany

**Keywords:** Cardiac magnetic resonance, Fast-strain-encoded MR (fast-SENC), Ischemic and non-ischemic cardiomyopathies, Late gadolinium enhancement, Heart failure, Hypertrophy, Myocarditis

## Abstract

**Aims:**

To compare the ability of left ventricular (LV) and right ventricular (RV) strain measured by fast-strain encoded cardiovascular magnetic resonance (CMR) (fast-SENC) with LV- and RV-ejection fraction for the diagnostic classification of patients with different stages of chronic heart failure (stages A-D based on American College of Cardiology/American Heart Association guidelines) due to non-ischemic cardiomyopathies.

**Methods:**

Our study population consisted of 276 consecutive patients who underwent CMR for clinical reasons, and 19 healthy subjects. Wall motion score index and non-infarct related late gadolinium enhancement (LGE), LV ejection fraction (LVEF) and RV ejection fraction (RVEF) and global LV- and RV-longitudinal (GLS) and circumferential strain (GCS) based on fast-SENC acquisitions, were calculated in all subjects. The percentage of LV and RV myocardial segments with strain ≤ − 17% (%normal LV and RV myocardium) was determined in all subjects.

**Results:**

LVEF and RVEF, LV-GLS, LV-GCS, RV-GLS, RV-GCS and %normal LV- and RV myocardium depressed with increasing heart failure stage (p < 0.001 for all by ANOVA). By multivariable analysis, %normal LV and RV myocardium exhibited closer associations to heart failure stages than LVEF and RVEF (r_partial_ = 0.79 versus r_partial_ = 0.21 for %normal LV myocardium versus LVEF and r_partial_ = 0.64 versus r_partial_ = 0.20 for %normal RV myocardium versus RVEF, respectively). Furthermore, %normal LV and RV myocardium exhibited incremental value for the identification of patients (i) with subclinical myocardial dysfunction and (ii) with symptomatic heart failure, surpassing that provided by LVEF and RVEF (ΔAUC = 0.22 for LVEF and ΔAUC = 0.19 for RVEF with subclinical dysfunction, and ΔAUC = 0.19 for LVEF and ΔAUC = 0.22 for RVEF with symptomatic heart failure, respectively, p < 0.001 for all). %normal LV myocardium reclassified 11 of 31 (35%) patients judged as having no structural heart disease by clinical and imaging data to stage B, i.e., subclinical LV-dysfunction.

**Conclusions:**

In patients with non-ischemic cardiomyopathy, %normal LV and RV myocardium, by fast-SENC, enables improved identification of asymptomatic patients with subclinical LV-dysfunction. This technique may be useful for the early identification of such presumably healthy subjects at risk for heart failure and for monitoring LV and RV deformation during pharmacologic interventions in future studies.

**Supplementary Information:**

The online version contains supplementary material available at 10.1186/s12968-021-00711-w.

## Introduction

Non-ischemic cardiomyopathies are a heterogeneous group of heart muscle diseases, which are frequently associated with genetic disorders [1]. Dilated cardiomyopathy (DCM) and hypertrophic cardiomyopathy (HCM) are the most common, accounting for a substantial proportion of cardiac mortality [2]. In addition, restrictive cardiomyopathies may be idiopathic or attributed to systemic disorders, such as amyloidosis [3]. The clinical course of non-ischemic cardiomyopathies is strongly heterogeneous, ranging from asymptomatic patients to those suffering from intractable heart failure [4–6]. Classical risk factors associated with an adverse outcome include age and male gender, New York Heart Association (NYHA) class, left atrial (LA) size and left ventricular (LV) and right ventricular (RV) dysfunction [7–9].

Due to its high intrinsic blood-to-tissue contrast and high spatial and temporal resolution, cardiovascular magnetic resonance imaging (CMR) is widely acknowledged as the central diagnostic tool for the characterization of cardiomyopathies. In addition, advanced CMR sequences, such as fast strain-encoded CMR (fast-SENC), provide quantification of longitudinal strain (LS) and circumferential strain (CS) while free breathing and without the need for an exogenous contrast agent [10]. We and others previously reported on the incremental value of fast-SENC for the diagnosis and risk stratification of patients with coronary artery disease (CAD) ([11, 12], and reviewed in[13]). However, data on fast-SENC in patients with non-ischemic cardiomyopathies are limited.

We therefore assessed the ability of LV and RV strain measured using fast-SENC CMR for the diagnostic classification of patients with different stages of chronic heart failure (A-D) due to non-ischemic cardiomyopathies. Classification in different stages of chronic heart failure was performed according to American College of Cardiology (ACC) and American Heart Association (AHA) guidelines [14]. In addition, we sought to compare the diagnostic value of LV and RV strain to RV ejection fraction (RVEF) and LV-ejection fraction (LVEF) and to non-infarct related late gadolinium enhancement (LGE) in those patients.

## Methods

### Study population

Our study population consisted of 276 consecutive patients and 19 healthy subjects, who underwent CMR between September 2017 and 2019 in the Marien Hospital Hamburg, Hamburg, Germany and in the German Heart Center, Berlin, Germany. Clinical indications for CMR included the assessment of myocardial function and LGE in patients at risk for or with symptoms of heart failure, the evaluation of LV-function and LGE for risk stratification of patients with non-ischemic cardiomyopathies or the diagnosis of specific cardiomyopathies, such as amyloidosis. CMR was performed as part of standard institutional protocols, unless one of the following contraindications to CMR was present: cardiac pacemaker or implantable cardioverter defibrillator, other non CMR compatible metallic implants, severe claustrophobia, obesity preventing patient entrance into the scanner bore, pregnancy and lactation. Chronic renal failure with an estimated glomerular filtration rate (eGFR) < 30 ml/min/1.73 m^2^ was considered as an exclusion criterion for administration of contrast agents. The presence of arterial hypertension, diabetes mellitus, hyperlipidemia, chronic kidney disease (eGFR < 60 ml/min/1,73m^2^), atrial fibrillation, left bundle branch block, prior myocardial infarction or known CAD were documented in all patients.

The diagnosis of non-ischemic cardiomyopathy was based on the 1995 World Health Organization/International Society and Federation of Cardiology criteria [15]. General exclusion criteria were: (i) significant CAD (defined as ≥ 50% diameter stenosis) by X-ray coronary angiography or computed tomography angiography, previous coronary revascularization, or myocardial infarction, (ii) more than moderate valvular disease, (iii) hypertensive heart disease and (iv) congenital abnormalities. DCM was defined by the presence of LV dilatation and impaired systolic function (LVEF ≤ 50%) [16], HCM was defined by the presence of unexplained LV hypertrophy (≥ 15 mm in adults or ≥ 13 mm in family member of HCM patients) in the presence of a nondilated cavity [17], whereas cardiac amyloidosis was diagnosed by Congo-red and immune-histologic staining using myocardial biopsy or defined by non-invasive imaging based on previously reported consensus criteria [18]. In addition, patients with isolated RV dysfunction due to pulmonary hypertension or arrhythmogenic RV dysplasia were excluded from analysis, as LV strain may be within normal ranges in such patients, whereas patients with sarcoidosis (n = 3) were excluded due to low sample size. Furthermore, patients with LV non-compaction and with LV-dysfunction due to cardiotoxicity were included with DCM. Patients with hypertensive heart disease and with myocarditis were excluded from analysis.

Nineteen healthy subjects without any CAD risk factors also underwent CMR for the acquisition of normal values for longitudinal strain (LS) and circumferential strain (CS) using fast-SENC. CMR examinations were performed by clinical indication and all patients provided signed informed consent. The use of patient data for research purposes was approved by the local ethics committees in accordance with the Declaration of Helsinki.

### Heart failure stages

Based on recommendations of the ACC and AHA recommendations, we identified the following 4 stages of heart failure [14, 19].

- Stage A: Patients at risk for developing heart failure due to the administration of cardiotoxic substances or familial predisposition but without evidence of symptomatic structural heart disease.

- Stage B: Patients with mild or moderate structural heart disorders (i.e. LV-hypertrophy, dilatation, myocardial fibrosis associated with non-infarct related LGE or other myocardial dysfunction either in the presence of preserved or reduced LVEF), who never experienced heart failure symptoms so far, corresponding to NYHA class I.

- Stage C: Patients with symptoms of heart failure symptoms such as dyspnea or fatigue, due to underlying structural heart disorders.

- Stage D: Patients with end-stage cardiac disease, NYHA class IV, despite intensive treatment.

Classification was performed by 2 experienced clinicians (HS and FA) based on demographic data, clinical presentation of heart failure symptoms and imaging including echocardiography and CMR.

### CMR baseline and late gadolinium enhancement (LGE) acquisitions

Standard CMR was performed on 1.5 T CMR scanners (Ingenia or Achieva, Philips Healthcare, Best, The Netherlands) equipped with cardiac phased array receiver coils. Cine images were obtained using a breath-hold balanced steady state free precession (bSSFP) sequence (typical slice thickness of 8 mm with 2 mm gap), employing retrospective electrocardiogram (ECG) gating in long axis planes (2-, 4- and 3-chamber views) and in contiguous short axis slices covering both ventricles, typically with 30 phases per cardiac cycle. After baseline acquisitions, LGE CMR was performed using a phase sensitive inversion recovery sequence and after the injection of 0.1 mmol/kg of Dotarem®-gadoterate meglumine (Gothia Medical AB, Billdal, Sweden) in three long axis and multiple short axis, covering the entire LV.

### Conventional CMR and LGE data analysis

All analyses were performed on a commercially available workstation (cvi 42, version 5.10, Circle Cardiovascular Imaging Inc., Calgary, Alberta, Canada). Results for ventricular volumes, LVEF, RVEF and LV mass were derived from short axis slices. Septal and lateral wall thickness and mitral annular plane systolic excursion (MAPSE) and tricuspid annular plane systolic excursion (TAPSE) were derived from the 4-chamber view. The presence of wall motion abnormalities and non-CAD related myocardial or pericardial LGE were semi-quantitatively evaluated.

Segmental wall motion was graded in all 17 segments using a 4-point scale:

- 1 = normal wall motion

- 2 = hypokinesia

- 3 = akinesia

- 4 = dyskinesia

Non-infarct LGE patterns were graded in all segments using a 2-point scale:

- 1 = normal

- 2 = abnormal non-infarct LGE

Subsequently, a wall motion score index was built for analysis by patients, by calculating the mean score in 17 myocardial segments, as recommended by the AHA [20]. For semiquantitative non-infarct LGE assessment, the number of segments was calculated in each patient. In addition, a semi-quantitative LGE score was used according to the % of segments with non-infarct related LGE thus ranging from 0% (no segments with LGE) up to 100% (all 17 segments with LGE) [21].

### Single heartbeat, Fast-SENC acquisitions

As described previously [10] fast-SENC is based on the acquisition of a high- and a low-tuning image with different frequency modulation. CS and LS in a range from 5% to − 30% were encoded using the fast-SENC sequence, with negative values representing myocardial contraction. A single heartbeat, ECG gated, fast-SENC variant with single-shot spiral readouts was used. Typical imaging parameters were as follows: field-of-view = 256 × 256 mm^2^, slice thickness = 10 mm, voxel size = 4 × 4 × 10 mm^3^, reconstructed resolution = 1 × 1 × 10 mm^3^, single-shot spiral readout with flip angle = 30°, effective echo time = 0.7 ms, repetition time = 12 ms, temporal resolution = 36 ms, typical number of acquired heart phases = 22, spectrally selective fat suppression, total acquisition time per slice < 1 s. Data were acquired in three long-axis (four-, three- and two-chamber) views, and three short-axis views of the LV (basal, mid-ventricular and apical).

### Fast-SENC data analysis

Circumferential strain was extracted from 3 long-axis views, whereas LS was extracted from the 3 short-axis images. The endocardial and epicardial borders were drawn at the end-systolic cardiac phase and then propagated throughout the cardiac cycle using an automatic tissue tracking algorithm. Tracking was verified and manually corrected if necessary.

For the LV, a 16-segment model was used for the LS (basal and mid anterior, anteroseptal, inferoseptal, inferior, inferolateral and anterolateral, respectively and apical anterior, septal, inferior and lateral) and a 21-segment model for the CS (basal and mid inferolateral and anteroseptal, respectively, apical lateral and anterior and the apical cap for the 3 chamber view; basal and mid inferoseptal and anterolateral, respectively, apical septal and lateral and the apical cap for the 4 chamber view and basal, mid and apical inferior and anterior, respectively and the apical cap for the 2 chamber view). For analysis by patients, global longitudinal strain (GLS) and global circumferential strain (GCS) were expressed as the average value of all 16 and 21 segments, respectively.

For the RV, a 6-segment model was used for LS (short axis basal and mid anterior, lateral, and inferior, respectively) and a 5-segment model for CS derived from the 4-ch and 3-ch views (basal and mid anterior and lateral, respectively and inferior lateral). For analysis by patients, RV GLS and RV GCS were expressed as the average value of all 6 and 5 segments, respectively.

Fast-SENC images were analyzed using the MyoStrain software (Myocardial Solutions, Inc., Morrisville, North Carolina, USA). Based on previous studies, reporting a cut-off value of − 17% for ‘normal’ myocardium in the LV [22, 23], we measured the total number of LV and RV segments per patient with LS or CS ≤ − 17%.

$${\text{LV or RV segments with normal myocardium}}\,= {\text{Number of LV or RV segments with LS and CS}} \le - 17\%$$

In addition, we calculated the percentage of LV or RV ‘normal’ myocardium in each patient, by considering the total number of segments LS ≤ − 17% (out of n = 16) and CS ≤ − 17% (out of n = 21) and by dividing this number by the total number of segments per patient (n = 37), as follows:

$$\%{\text{Normal myocardium}}= \frac{{{\text{Segments with circumferential and longitudinal strain }} \le - 17\%}}{37}$$

### Statistical analysis

Continuous variables were expressed as mean ± standard deviation for parametric or as median with interquartile range for nonparametric variables. For continuous variables, differences between two groups were compared using Students´ t-test (if normally distributed) or Mann Whitney U test (if not normally distributed). Categorical variables were expressed as counts and percentages and compared by Chi-square test or Fisher's exact test, respectively. Receiver operating characteristics (ROC) analysis was used for the classification of patients with different heart failure stages and pairwise comparisons of areas under the curves were assessed. Comparison of areas under the curves (AUC) was performed using the DeLong method. Based on ROC derived cut-off values for %normal myocardium, patients with abnormal strain in the presence of normal functional data were reclassified, depending on their clinical presentation from stage A to stage B (asymptomatic myocardial dysfunction). Univariate and multivariable logistic regression analysis was performed to calculate odds ratios (OR) and 95% confidence intervals (CI). Furthermore, hierarchic logistic regression models were used to assess the incremental value of %normal LV/RV myocardium and LVEF/RVEF and to clinical variables (age and NYHA class). The ANOVA test was used for unpaired, parametric and the Kruskal–Wallis test for unpaired, nonparametric testing [24]. In addition, Scheffé tests were used for post-hoc analysis [25]. Furthermore, logistic regression analysis was performed to test the association between mean segmental CS and LS for the presence or absence of non-infarct related LGE after adjustment for intra-cluster correlation for segments within the same subjects (mixed effects analysis). Inter- and intra-observer variabilities for strain values were assessed by repeated analysis of 40 randomly selected patients and were calculated as the ratio of the standard deviation to the mean. Furthermore, Bland–Altman plots are provided with strain measures. In addition, observer agreement between 2 experienced clinicians (HS & FA) for the classification of heart failure was assessed in 50 randomly selected cases using weighted κ-statistics. Readings were separated by 8 weeks to minimize recall bias. MedCalc (version 18.11.6; MedCalc, Ostend, Belgium, 2019) was used throughout.

## Results

### Clinical characteristics and CMR baseline data

Data were analyzed in 212 patients with DCM, 34 with HCM and 30 patients with cardiac amyloidosis and in 19 healthy subjects. Clinical and baseline CMR characteristics of healthy subjects and patients are illustrated in Table 1. Representative fast-SENC images are provided in Additional file 1: Figure S1. Significant differences were present for clinical and for CMR variables with different cardiomyopathies. Patients with amyloidosis were older and demonstrated more advanced NYHA class symptoms and lower strain values. In addition, HCM patients demonstrated preserved LVEF and RVEF in the presence of reduced strain values.

**Table 1:** Clinical and CMR data of healthy subjects and patients with cardiomyopathies

Parameters	Heathy subjects (n = 19)	Dilated cardiomyopathy (n = 212)	Hypertrophic cardiomyopathy (n = 34)	Cardiac amyloidosis (n = 30)	p-values*
Clinical and demographic data					
Age	30 ± 7	53 ± 18	58 ± 14	69 ± 12	< 0.001
Female gender; n (%)	8 (42%)	87 (41%)	16 (47%)	12 (40%)	NS
Arterial hypertension	0 (0%)	93 (44%)	28 (82%)	19 (63%)	< 0.001
Hyperlipidemia	0 (0%)	70 (33%)	16 (47%)	14 (47%)	0.02
Diabetes mellitus	0 (0%)	26 (12%)	8 (24%)	4 (13%)	NS
Atrial fibrillation	0 (0%)	22 (10%)	3 (9%)	11 (37%)	< 0.001
Left bundle branch block	0 (0%)	20 (9%)	3 (9%)	0 (0%)	NS
Body mass index (kg/m^2^)	23 ± 7	26 ± 5	29 ± 5	26 ± 3	0.01
NYHA class	NA	2.1 ± 0.8	2.3 ± 0.7	2.7 ± 0.6	0.001
Baseline CMR data					
LVEF (%)	60.1 ± 5.2	48.0 ± 13.2	55.9 ± 9.1	47.6 ± 10.2	0.002
LV End-diastolic volume (ml)	137 ± 27	190 ± 27	166 ± 41	175 ± 40	NS
LV End-diastolic volume index (ml/m^2^)	76 ± 12	95 ± 34	81 ± 17	88 ± 20	< 0.05
LV End-systolic volume (ml)	54 ± 14	103 ± 72	73 ± 31	94 ± 35	< 0.05
LV End-systolic volume index (ml/m^2^)	30 ± 7	52 ± 34	36 ± 14	47 ± 18	< 0.05
LV Stroke volume (ml)	84 ± 15	86 ± 22	93 ± 23	82 ± 20	NS
LV Stroke volume index (ml/m^2^)	47 ± 7	44 ± 10	46 ± 10	41 ± 10	NS
Septal wall thickness (mm)	7.4 ± 1.7	9.5 ± 2.5	17.3 ± 6.1	16.0 ± 2.8	< 0.001
Infero-lateral wall thickness (mm)	5.2 ± 1.4	6.9 ± 1.8	8.9 ± 3.0	11.7 ± 3.6	< 0.001
LV mass (g)	75 ± 12	119 ± 39	160 ± 48	175 ± 51	< 0.001
LV mass index (g/m^2^)	42 ± 7	60 ± 17	78 ± 19	88 ± 24	< 0.001
MAPSE (mm)	13 ± 3	10 ± 3	9 ± 2	7 ± 3	< 0.001
RVEF (%)	57.7 ± 4.5	52.2 ± 10.1	58.1 ± 6.6	49.2 ± 12.3	0.001
TAPSE (mm)	24 ± 6	21 ± 6	21 ± 6	17 ± 5	0.001
Ascending aorta (mm)	22 ± 3	30 ± 6	33 ± 5	35 ± 4	< 0.001
Aortic arch (mm)	17 ± 2	23 ± 3	25 ± 4	26 ± 3	< 0.001
Descending aorta (mm)	17 ± 2	22 ± 3	24 ± 4	25 ± 4	< 0.001
Left atrial diameter (mm)	27 ± 4	37 ± 9	41 ± 9	43 ± 10	< 0.001
Left atrial area index (cm^2^/m^2^)	11 ± 2	14 ± 3	15 ± 6	16 ± 4	< 0.001
Pulmonary trunk diameter (mm)	22 ± 2	26 ± 4	26 ± 3	29 ± 4	< 0.01
Wall motion score index	1.0 ± 0.0	1.4 ± 0.5	1.2 ± 0.5	1.6 ± 0.5	0.01
%non-infarct related LGE segments	0	20 ± 19	22 ± 12	66 ± 43	< 0.001
CMR global strain data					
LV global longitudinal strain (%)	− 21.3 ± 1.3	− 17.2 ± 3.8	− 15.4 ± 3.3	− 12.1 ± 4.1	< 0.001
LV global circumferential strain (%)	− 20.5 ± 1.1	− 17.2 ± 3.0	− 16.4 ± 2.0	− 14.0 ± 2.8	< 0.001
RV global longitudinal strain (%)	− 21.2 ± 1.1	− 18.8 ± 2.8	− 17.7 ± 2.8	− 14.8 ± 4.0	< 0.001
RV global circumferential strain (%)	− 19.2 ± 1.3	− 16.8 ± 3.0	− 15.7 ± 1.7	− 13.6 ± 3.4	< 0.001

^*^p-values are reported for differences between patients with cardiomyopathies, excluding healthy subjects

Data are presented as means ± standard deviations or as proportions. NA indicates not applicable; *LVEF* left ventricular ejection fraction, *RVEF* right ventricular ejection fraction, *NYHA* New York Heart Association, *MAPSE* mitral annular plane systolic excursion, *TAPSE* tricuspid annular plane systolic excursion

Control subjects exhibited global LV values of − 21.3 ± 1.3% and − 20.5 ± 1.1% for GLS and GCS and − 21.2 ± 1.1% and − 19.2 ± 1.3% for RV GLS and GCS, respectively. Mean LVEF and RVEF was 60.1 ± 5.2% and 57.7 ± 4.5%, respectively.

### Correlations between LVEF, wall motion score index, non-infarct related LGE and strain

**Fig. 1:**
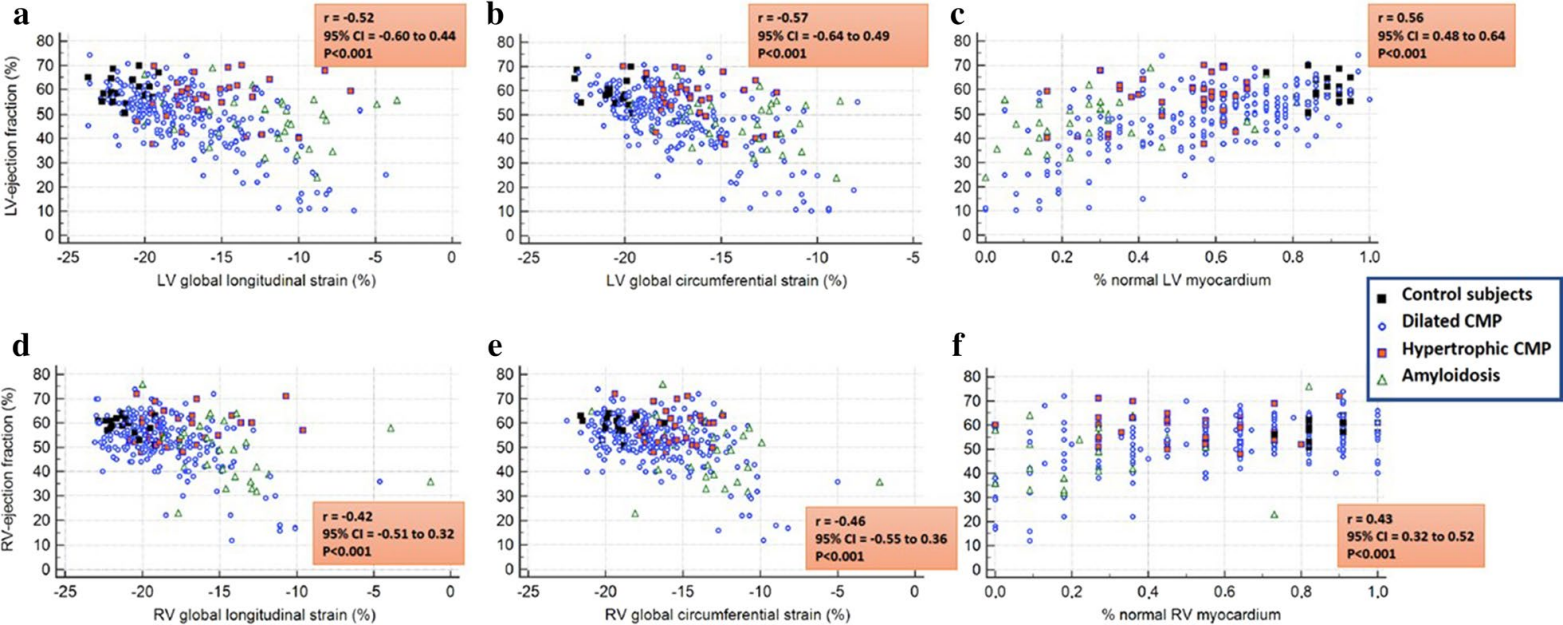
Moderate correlations were found between left ventricular (LV) ejection fraction (LVEF) (%) with LV global longitudinal strain (GLS) and global circumferential strain (GCS) and with %normal myocardium for the LV (**a**–**c**) and right ventricle (RV) (**d**–**f**)

Moderate to poor correlations were found between LVEF with GLS and GCS and with %normal myocardium for both the LV (Fig. 1a–c) and the RV (Fig. 1d–f).

**Fig. 2:**
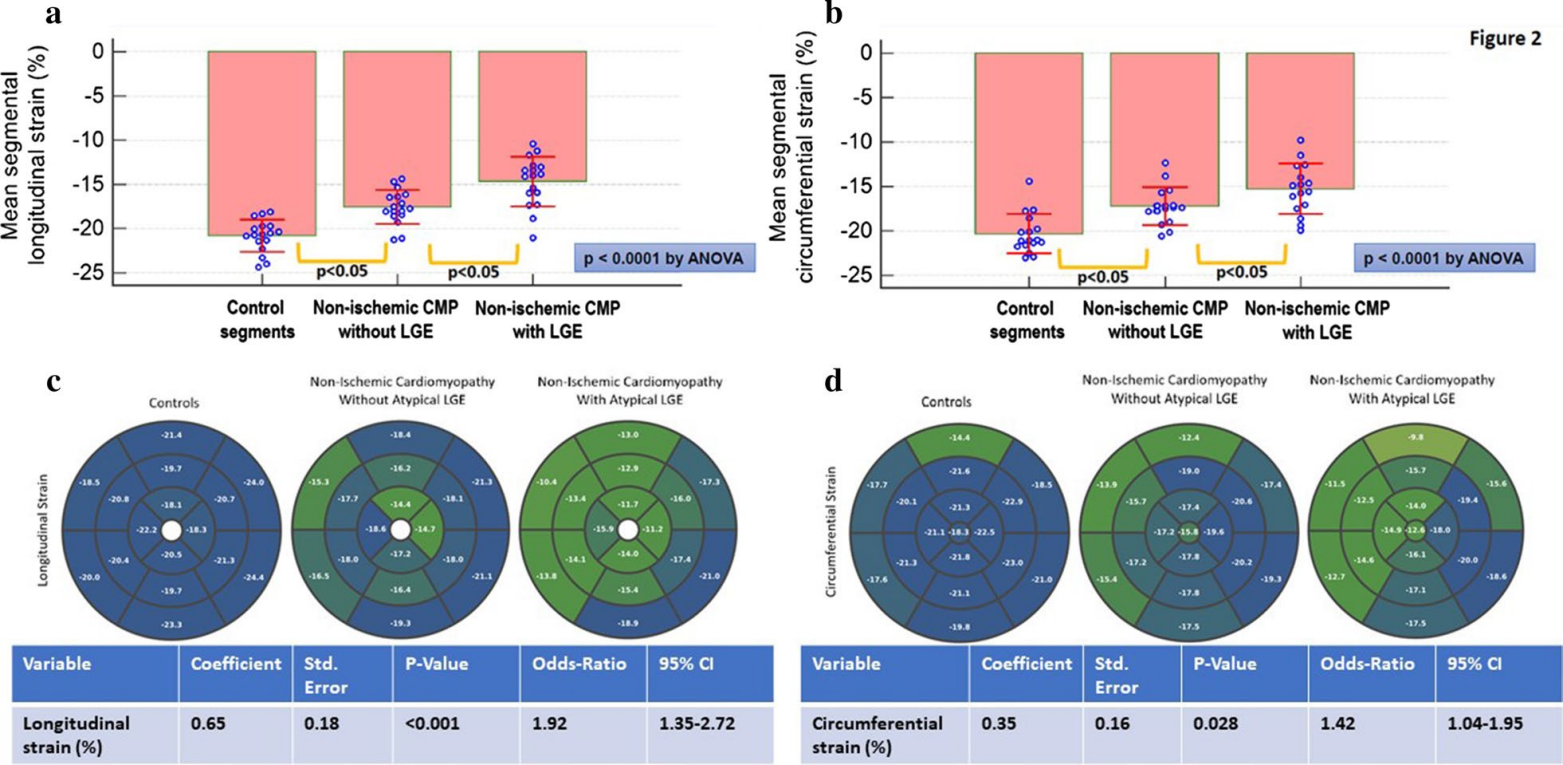
Strain and non-infarct related late gadolinium enhancement (LGE) analysis by segments: Segments of controls showed the highest longitudinal and circumferential strain values, followed by segments of patients with non-ischemic cardiomyopathy without non-infarct related LGE and then by segments of patients with non-ischemic cardiomyopathy with non-infarct related LGE (**a**, **b**). For segmental analysis, the standard deviations of mean segmental strain values are provided. The corresponding polar maps of regional strain values in healthy subjects and in segments of patients with non-ischemic cardiomyopathy without and with LGE, as well as logistic regression analysis for the ability of mean longitudinal and circumferential segmental strain to predict the presence of non-infarct related LGE in a given myocardial segment, are provided in **c**, **d**

Analyzing by segments, controls showed higher LS and CS compared to non-ischemic cardiomyopathy without LGE, followed by non-ischemic cardiomyopathy with LGE (Fig. 2a, b). The corresponding polar maps of regional strain values in these 3 categories as well as logistic regression analysis for the ability of mean LS and CS strain to predict the presence of non-infarct related LGE in a given myocardial segment is provided in Fig. 2c, d.

Furthermore, moderate correlations were found between LVEF and RVEF and with %normal LV and RV myocardium with wall motion and non-infarct related LGE (Table 2).

**Table 2:** Correlations between LVEF and RVEF, %normal LV & RV myocardium and wall motion and non-infarct related LGE

Parameters	LVEF	RVEF	%normal LV myocardium	%normal RV myocardium	Wall motion score index	% LV segments with non-infarct related LGE
LVEF	1.0	0.44 p < 0.001	0.56 p < 0.001	0.50 p < 0.001	− 0.49 p < 0.001	− 0.26 p < 0.001
RVEF	MA	1.0	0.49 p < 0.001	0.43 p < 0.001	− 0.49 p < 0.001	− 0.20 p = 0.001
%normal LV myocardium	MA	MA	1.0	0.82 p < 0.001	− 0.68 p < 0.001	− 0.42 p < 0.001
%normal RV myocardium	MA	MA	MA	1.0	− 0.58 p < 0.001	− 0.47 p < 0.001
Wall motion score index	MA	MA	MA	MA	1.0	0.34 p < 0.001
% LV segments with non-infarct related LGE	MA	MA	MA	MA	MA	1.0

*MA* mentioned above, *LGE* late gadolinium enhancement

### Myocardial strain by heart failure stages

**Fig. 3:**
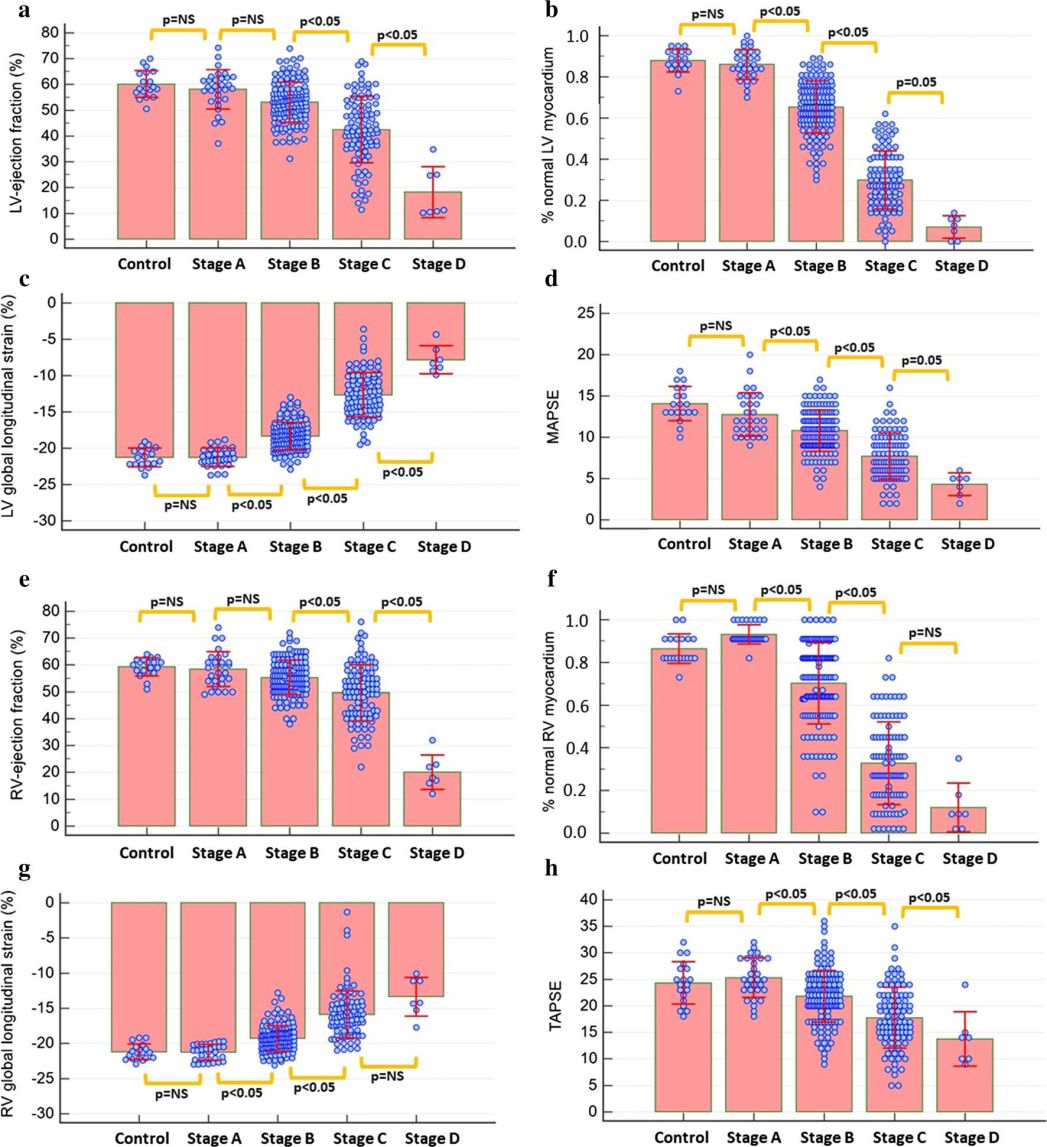
LV-ejection fraction (LVEF) and RV-ejection fraction (RVEF), global circumferential strain (GCS), %normal LV and RV myocardium as well as mitral annular plane systolic excursion (MAPSE) and tricuspid annular plane systolic excursion (TAPSE) significantly decreased with increasing heart failure stage (**a**–**h**)

Both LVEF and RVEF, GLS, %normal LV and RV myocardium and MAPSE and TAPSE gradually decreased with increasing heart failure stage (Fig. 3a–h). Especially %normal LV and RV myocardium already decreased in stage B patients, whereas other parameters, such as LV and RV-ejection fraction decreased with later heart failure stages.

The early deterioration of %normal LV strain with stage B patients remained with DCM, HCM and amyloidosis, whereas %normal RV strain also diminished early with stage B patients in DCM, but not in HCM or cardiac amyloidosis (Additional file 2: Figure S2).

### Uni- and multivariable analysis

Age, NYHA class, LVEF, RVEF, non-infarct related LGE as well as and LV and RV GLS and GCS and % normal LV and RV myocardium were predictive of clinical heart failure stages.

By multivariable analysis, %normal LV and RV myocardium exhibited the highest association with heart failure stages, surpassing that provided by clinical parameters and conventional CMR variables (Table 3).

**Table 3:** Uni- and multivariable analysis for the prediction of heart failure stages. Absolute values are reported for all correlation coefficients

Variables	r_partial_	p-values	r_partial_	p-values	r_partial_	p-values	r_partial_	p-values	r_partial_	p-values
	Univariable		Multivariable model LV#1		Multivariable model LV#2		Multivariable model RV#1		Multivariable model RV#2	
Age (yrs.)	*0.49*	< *0.001*	*0.28*	< 0.001	*0.16*	< 0.05	*0.33*	< 0.001	*0.27*	< 0.001
Arterial hypertension	0.25	< 0.001	–	–	–	–	–	–	–	–
Diabetes mellitus	0.17	< 0.01	–	–	–	–	–	–	–	–
Chronic kidney disease	0.23	< 0.001	–	–	–	–	–	–	–	–
Atrial fibrillation	0.18	< 0.01	–	–	–	–	–	–	–	–
Left bundle branch block	0.22	< 0.001	–	–	–	–	–	–	–	–
NYHA class	*0.58*	< 0.001	*0.17*	< 0.01	*0.21*	< 0.01	*0.25*	0.003	*0.24*	< 0.001
LVEF (%)	− *0.59*	< 0.001	− *0.29*	< 0.001	− *0.21*	< 0.001	–	–	–	–
RVEF (%)	− 0.51	< 0.001	–	–	–	–	− *0.22*	< 0.001	− *0.20*	< 0.01
MAPSE (mm)	− 0.68	< 0.001	− 0.12	NS	− 0.04	NS	–	–	–	–
TAPSE (mm)	− 0.52	< 0.001	–	–	–	–	− *0.15*	0.02	− 0.06	NS
LV mass index	0.63	< 0.001	–	–	–	–	–	–	–	–
Wall motion score index	0.70	< 0.001	–	–	–	–	–	–	–	–
%non-infarct LGE segments	0.44	< 0.001	–	–	–	–	–	–	–	–
LV global longitudinal strain (%)	*0.87*	< 0.001	–	–	–	–	–	–	–	–
LV global circumferential strain (%)	*0.87*	< 0.001	*0.70*	< 0.001	-	-	–	–	–	–
RV global longitudinal strain (%)	*0.69*	< 0.001	–	–	–	–	*0.48*	< 0.001	–	–
RV global circumferential strain (%)	*0.76*	< 0.001	–	–	–	–	–	–	–	–
%normal LV myocardium	− *0.92*	< 0.001	–	–	− *0.79*	< 0.001	–	–	–	–
%normal RV myocardium	− *0.81*	< 0.001	–	–	–	–	–	–	− *0.64*	< 0.001

NYHA indicates New York Heart Association and LGE, late gadolinium enhancement and *GFR* glomerular filtration rate. Chronic Kidney disease was defined as (GFR < 60/min/1.73m^2^)

### Identification of asymptomatic patients with subclinical LV-dysfunction (Stage B), and of patients with clinical disease and with refractory heart failure

**Fig. 4:**
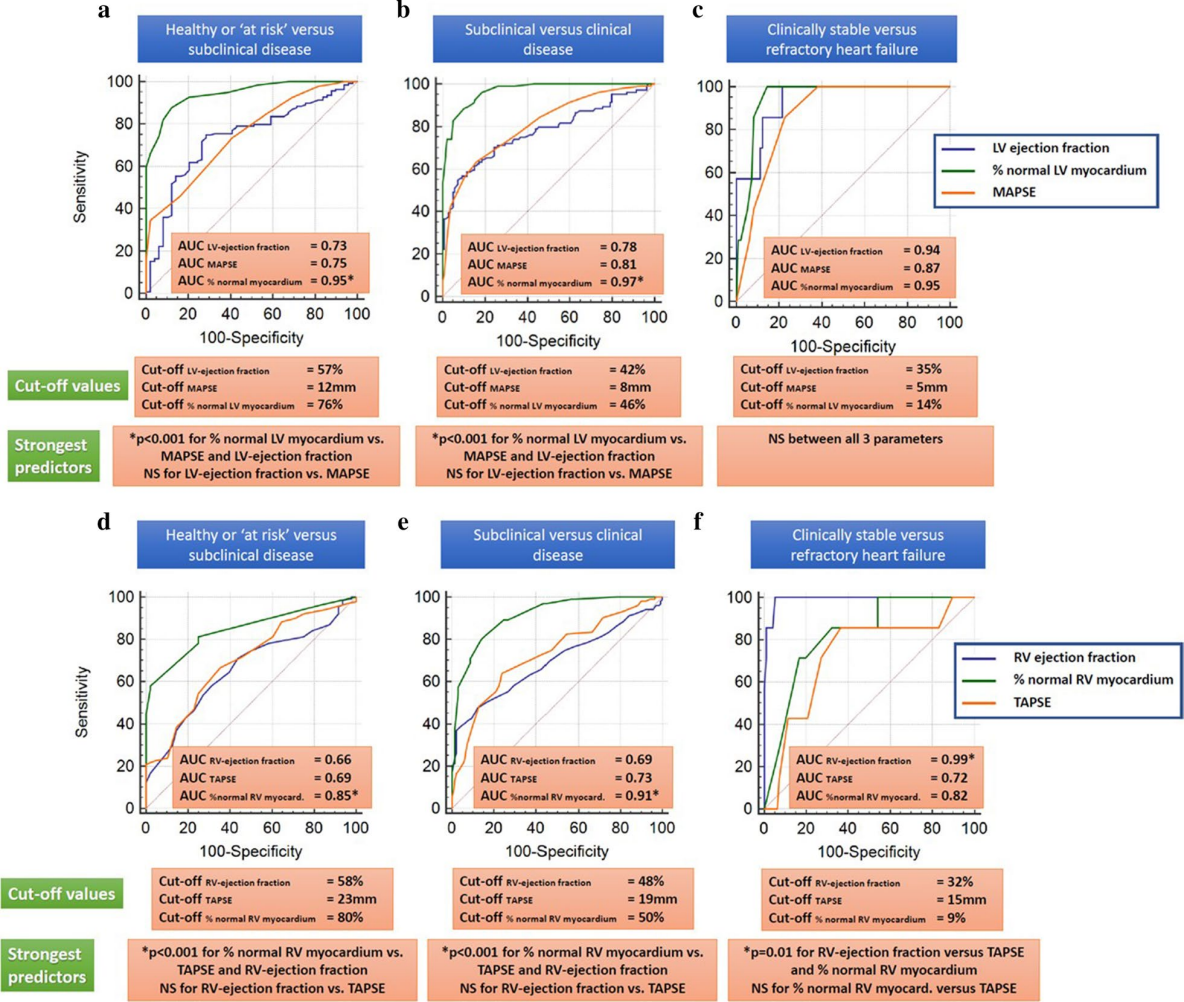
%normal LV myocardium exhibited incremental value for the identification of patients with subclinical LV-dysfunction and for the differentiation of clinical versus subclinical heart failure, surpassing the value of LVEF and MAPSE. All 3 parameters were equal for the identification of patients with refractory stage D heart failure (**a**–**c**). %normal RV myocardium exhibited incremental value for the identification of patients with subclinical LV-dysfunction and for the differentiation of clinical versus subclinical heart failure, surpassing the value of RVEF and TAPSE. RVEF exhibited the highest accuracy for the identification of patients with refractory heart failure (**d**–**f**)

%normal LV and RV myocardium exhibited incremental value for the identification of patients with subclinical myocardial dysfunction and for the differentiation of clinical versus subclinical heart failure, surpassing the values of LVEF and RVEF (ΔAUC = 0.22 for the LV and ΔAUC = 0.19 for the RV, and ΔAUC = 0.19 for the LV and ΔAUC = 0.22 for the RV, respectively, p < 0.001 for all), (Fig. 4a, b and d–e).

All 3 LV-parameters, on the other hand, performed similarly for the identification of patients with refractory stage D heart failure (Fig. 4c), whereas RVEF, exhibited the highest potential for the identification of patients with refractory heart failure (Fig. 4f).

LV and RV specific threshold values for the differentiation between healthy controls and stage A versus subclinical dysfunction and between asymptomatic and symptomatic heart failure are summarized in Table 4. In addition, the accuracy of LV and RV functional parameters and %normal myocardium for the differentiation of patients between the asymptomatic stage B and symptomatic stages C/D are shown in Table 5.

**Table 4:** LV and RV specific threshold values for the differentiation between healthy controls and stage A versus subclinical dysfunction and between asymptomatic and symptomatic heart failure

**Parameters**	**Healthy or ‘at risk’ versus subclinical disease**	**Subclinical versus symptomatic disease**	**Clinically stable versus refractory heart failure**
LVEF (%)	57%	42%	35%
%normal LV myocardium	76%	46%	14%
MAPSE (mm)	12	8	5
RVEF (%)	58%	48%	32%
%normal RV myocardium	80%	50%	9%
TAPSE (mm)	23	19	15

**Table 5:** Areas under the curve with their corresponding 95% confidence intervals for the differentiation between asymptomatic and symptomatic heart failure in patients with DCM, HCM and cardiac amyloidosis

**Parameters**	**All non-ischemic cardiomyopathies**	**DCM**	**HCM**	**Cardiac amyloidosis**
LVEF (%)	0.78 (0.72 to 0.83)	0.83 (0.77 to 0.89)	0.67 (0.49 to 0.82)	0.59 (0.39 to 0.76)
%normal LV myocardium	0.97 (0.94 to 0.99)	0.97 (0.93 to 0.99)	0.94 (0.80 to 0.99)	0.98 (0.85 to 1.00)
RVEF (%)	0.69 (0.63 to 0.75)	0.71 (0.63 to 0.77)	0.57 (0.39 to 0.73)	0.80 (0.62 to 0.92)
%normal RV myocardium	0.91 (0.87 to 0.95)	0.94 (0.89 to 0.97)	0.69 (0.51 to 0.84)	0.93 (0.77 to 0.99)

**Fig. 5:**
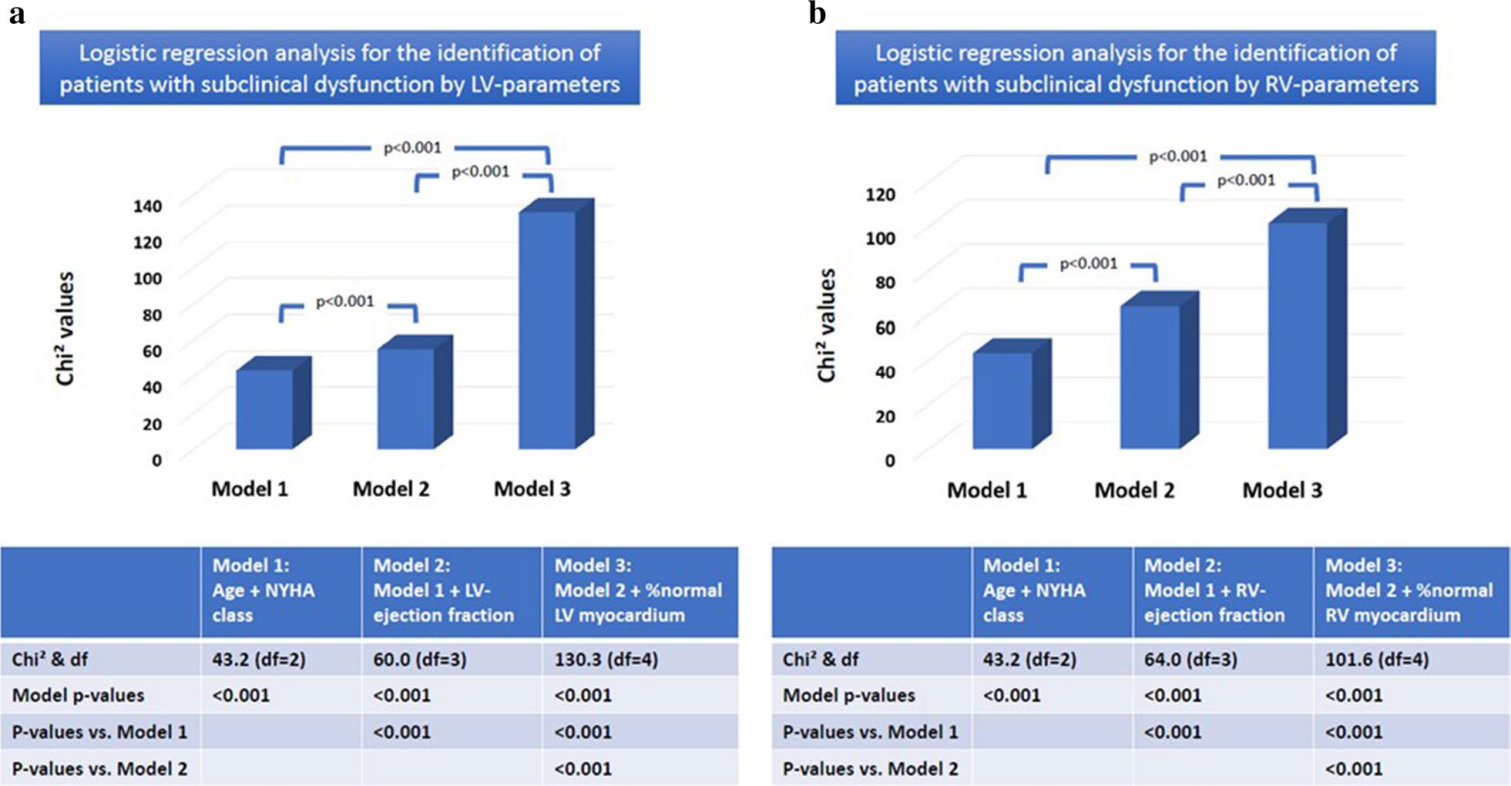
Logistic regression analysis demonstrated the incremental predictive role of %normal LV (**a**) and RV (**b**) myocardium for the identification of patients with subclinical myocardial dysfunction independent of age, NYHA class and LVEF/RVEF

Logistic regression analysis confirmed the incremental predictive role of %normal LV and RV myocardium for the identification of patients with subclinical myocardial dysfunction independent of age, NYHA class and LVEF and RVEF (Fig. 5a, b).

**Fig. 6:**
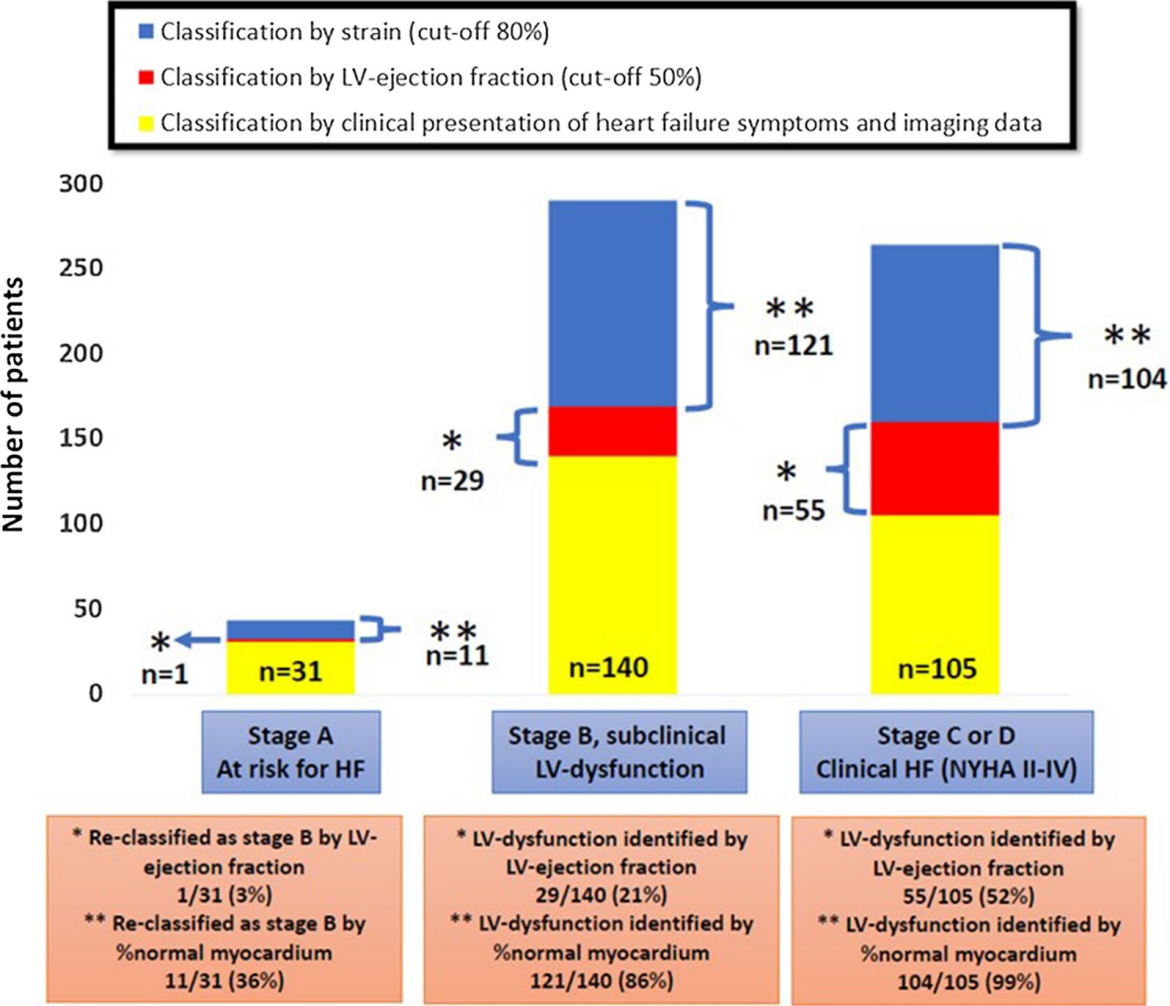
Eleven of 31 (36%) patients at stage A, i.e., at risk for heart failure but with no structural or functional abnormalities where re-classified by %normal LV myocardium to stage B, i.e., subclinical LV-dysfunction. LV-dysfunction was detected by LVEF (threshold = 50%) and %normal LV myocardium (threshold = 80%) in 29/140(21%) and 121/140(86%) of patients with subclinical disease and in 55/105(52%) and 104/105(99%) of patients, respectively with overt heart failure

%normal LV myocardium re-classified 11 of 31(36%) patients without any structural or functional abnormalities from stage A to B. In addition, %normal LV myocardium detected functional abnormality in significantly more patients at stage B and C than LVEF (29/140(21%) versus 121/140(86%) and 55/105(52%) versus 104/105(99%), respectively, p < 0.001 for both), (Fig. 6).

### Observer variabilities

**Fig. 7:**
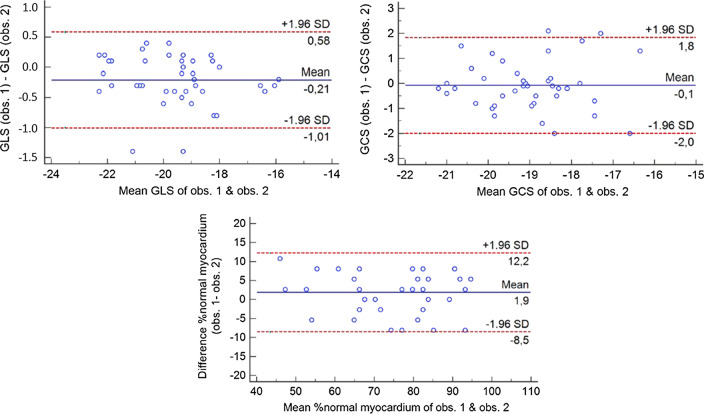
Bland–Altman plots, exhibiting narrow limits of agreement for global longitudinal strain (GLS) and global circumferential strain (GCS), and for %normal myocardium

Intra- and interobserver coefficients of variation for global strain were 1.1% and 1.2% for GLS, 2.4% and 2.2% for GCS and 4.2% and 4.9% for %normal myocardium, respectively. The corresponding Bland–Altman plots are provided in Fig. 7. Agreement regarding the assessment of heart failure stages was good weighted κ = 0.89, SE = 0.04, 95%CI = 0.80–0.98.

## Discussion

The main findings of the present study are:

1. Single heartbeat fast-SENC allows for comprehensive and reproducible assessment of CS and LS LV and RV strain in healthy subjects and in patients with non-ischemic cardiomyopathies of different etiologies and at different clinical heart failure stages.
2. LV and RV strain parameters exhibit moderate to poor correlations with MAPSE, TAPSE, wall motion and non-infarct related LGE and with LVEF and RVEF.
3. %normal LV and RV myocardium are the most robust parameters for the identification of patients with subclinical myocardial dysfunction exhibiting incremental value to LVEF/RVEF. Thus, %normal LV myocardium using a ROC derived cut-off value of 80% re-classifies ~1/3 of patients without any structural or functional abnormalities (normal functional data) from stage A to stage B, i.e., subclinical LV-dysfunction, whereas it detects functional abnormality in significantly more patients at stage B than LVEF (86% versus 21%, p < 0.001).
4. RVEF appears to be the most robust parameter for identifying patients with refractory heart failure.

### Heart failure due to non-ischemic cardiomyopathies

Heart failure is a progressive health disorder and a major cause of morbidity and mortality in the Western world, resulting in millions of deaths and hospitalizations annually [26]. Thus, the number of individuals affected by symptomatic heart failure is expected to increase in the US up to 8 million people by 2030, causing an immense increase of the total healthcare costs to over $50 billion [27, 28]. The magnitude of this socioeconomic problem has been emphasized by the ACC/AHA, which therefore developed a staging classification system of heart failure, based on cardiac risk factors, evidence of cardiac involvement and clinical manifestations [14].

Non-ischemic cardiomyopathies are heart muscle diseases, which significantly contribute to morbidity and mortality in developed countries with a reported 10-year heart failure mortality rate of over 40% [2, 7]. Non-ischemic DCM is a relatively common, genetically associated heart muscle disorder with a prevalence of 1:2,500 adults. This condition is associated with significant mortality due to progressive heart failure and sudden cardiac death and remains the leading indication for heart transplantation [7, 29]. HCM on the other hand, is a genetic disorder, which is characterized by hypertrophy, disarray of myocardial fibers and regional fibrosis [30]. Like DCM, substantial variability is observed in terms of phenotypic expression and natural progression of the disease. Notably, sudden cardiac death may occasionally be the initial manifestation in otherwise asymptomatic young patients, whereas most patients experience a benign clinical trajectory with relatively low events rates [31, 32]. In addition, amyloidosis is a systemic disorder with leads to abnormal protein (amyloid) deposition in the heart muscle, causing increased myocardial stiffness [33].

### Previous CMR studies

CMR provides unobstructed views of the heart in any desired plane and is widely recognized as the gold standard strategy for the truly tomographic assessment of LV-function with high accuracy and excellent reproducibility [34]. Previous studies demonstrated the versatility of CMR for the diagnostic work-up of patients with DCM, HCM and cardiac amyloidosis, enabling the assessment of LV-function, mass and LGE, the latter being an independent prognostic marker in such patients [5, 35, 36]. Fewer studies however, focused on the role of myocardial strain for the diagnostic classification and risk stratification of patients with non-ischemic cardiomyopathies. In this regard, we and others previously demonstrated that myocardial strain based on feature tracking is the most robust predictors of cardiac events in patients with non-ischemic cardiomyopathies, surpassing the value of LVEF [37, 38]. In addition, reduced GLS and GCS was associated with increased fibrosis and with poor cardiac outcomes such as ventricular arrhythmias, heart failure and cardiovascular death in HCM patients [39, 40]. Similarly, myocardial strain was predictive of mortality in patients with cardiac amyloidosis. However, only a few studies are available investigating the value of myocardial strain across a broad spectrum of ambulatory patients with heart failure due to non-ischemic cardiomyopathies.

In addition, few studies have so far focused on RV functional assessment in heart failure patients, which may be partially attributed to its complex anatomic structure, posing challenges for assessment of RV morphology and structure [41]. However, RV dysfunction and remodeling seems to be an important component, particularly at later stages of heart failure. In this regard, previous studies in patient cohorts of mixed ischemic and non-ischemic etiology suggested that RVEF is a determinant of exercise capacity and outcomes [42, 43]. Furthermore, Gulati et al. recently suggested that RV-dysfunction is a major predictor of poor clinical outcomes in patients with non-ischemic cardiomyopathy, such as all-cause mortality and need for heart transplantation [44].

### Our results and clinical implications

We demonstrated that myocardial strain by the fast-SENC sequence can aid the accurate and reproducible assessment of LV and RV function, with moderate to poor associations to conventional CMR variables such as LV/RV function, wall motion score index and non-infarct related LGE, whereas strong associations were observed with clinical heart failure stages by ACC and AHA guidelines [14]. The repeatability of strain measures by fast-SENC is clinically acceptable, as demonstrated by low bias and good repeatability coefficients using Bland–Altman plots. Normal LV and RV myocardium with cut-off values of ~ 80%, exhibited substantially higher accuracy for the identification of patients with subclinical myocardial dysfunction at stage B than LVEF and RVEF and other conventional CMR variables. The corresponding cut-off values for LVEF and RVEF derived by ROC analysis were 57% and 58%, respectively, which is within the range of normal values further highlights the difficulty of ejection fraction measures to depict subtle functional alterations in such patients. In addition, the clinical LVEF cut-off of 50% detected only in 21% of patients with subclinical heart failure and 52% of patients with overt heart failure. Fast-SENC using a cut-off value of < 80% normal myocardium on the other hand, detected subclinical myocardial dysfunction already in ~ 1/3 of patients at risk for heart failure without any structural heart disease and with normal functional data, in 86% of patients with subclinical disease and in 99% patients with symptomatic heart failure. Especially, the detection of subclinical LV-dysfunction in patients with no structural abnormalities and in patients at stage B with normal ejection fraction and minor structural abnormalities, such as non-infarct related LGE or mild LV-hypertrophy, highlights the value and potential of this tool for clinical risk stratification in early, subclinical heart failure stages (Fig. 5).

In this regard, LV strain exhibited higher accuracy for the differentiation of early stages of heart failure, whereas RV strain and functional data appeared more robust for the identification of patients with refractory heart failure (Fig. 3). For this reason, we focused on LV strain changes for the reclassification from stage A to B.

Furthermore, LV and RV strain showed higher accuracy for the identification of symptomatic disease (shift from stage B to C) in most subtypes of cardiomyopathy compared to traditional functional data. RV strain exhibited high accuracy for the detection of symptomatic disease in DCM and amyloidosis, whereas the accuracy was moderate for HCM (Table 5).

The early identification of such patients who do not exhibit clinical heart failure symptoms and are considered ‘healthy’ but already have reduced LV and RV myocardial performance using fast-SENC may have vast medical and socioeconomic implications. Thus, such individuals may profit from early risk factor control and ‘cardioprotection’ using pharmacologic treatment for example with ß-blockers and angiotensin-converting enzyme inhibitor, angiotensin receptor blockers or neprilysin inhibitors. This hypothesis of course merits further investigation in future interventional trials. The detection of subclinical LV and RV dysfunction by fast-SENC in the vast majority of such patients with normal traditional functional parameters however, already underscores the importance of strain measures in this vulnerable group of patients, who are clinically unremarkable, but are already at high risk to develop symptomatic heart failure [19]. At later stages of heart failure, RV function appeared to be the most robust parameter for identifying patients with severe and non-retractable heart failure, which agrees with previous reports [44]. It should be noted, that the cut-off value of myocardial strain ≥ − 17% was selected based on previous studies [22, 23], but is quite close to the mean, minus 2 standard deviations of normal global strain values in our healthy subjects.

### Physiologic and technical considerations

LV- and RV function are not only determined by radial contraction, but also by longitudinal and circumferential components [45], the latter 2 components being less considered during LVEF and RVEF assessment. Based on previous experimental data, longitudinal and circumferential function are more sensitive surrogate markers of contractile function [46]. Thus, it is not surprising that %normal LV and RV myocardium based on mean values of CS and LS were more sensitive markers for the identification of patients with subclinical LV-dysfunction compared to LVEF and RVEF.

Most of the previous studies used feature tracking for the assessment of LV GLS and GCS in patient cohorts with non-ischemic cardiomyopathies [37–39]. From a technical point of view, feature tracking can indeed be applied to standard CMR cine sequences and therefore obviate the need for dedicated pulse sequences for the quantification of myocardial strain. However, the feature tracking algorithm may pose several difficulties, which limit its reproducibility, as underscored in recent studies [47]. Other strain techniques, such as fast-SENC, may perform better than feature tracking since fast-SENC allows for a single heart-beat and more comprehensive evaluation of global myocardial strain with high reproducibility [10].

## Limitations

Some limitations need to be mentioned. Our study does not include outcome data. However, previous studies have underscored the strong prognostic implications of heart failure stages, as defined by the recommendations of the ACC/AHA [19]. In addition, we investigated patients with different forms of non-ischemic cardiomyopathy, including DCM, HCM and cardiac amyloidosis, which all have a different pathophysiologic background and therefore differently altered cardiac mechanics. However, the association between %normal LV and RV myocardium, which is the key message of our study, remained significant with DCM, HCM and amyloidosis (Additional file 2: Figure S2). In addition, a major limitation with our study is the lack of quantification analysis with non-infarct related LGE. This may have introduced biases, in favor of strain quantification over LGE, which was graded by visual criteria. However, since we included patients with amyloidosis, where LGE quantification is challenging due to the diffuse nature of LGE in these patients and because the nulling of the LV cavity is often strongly altered, semiquantitative analysis was provided for all patients of our cohort, similar to previously published methodology [21]. In addition, the focus was set on chronic heart failure and patients with acute myocarditis were excluded, since follow-up CMR studies would have been necessary with this clinical entity, which were not considered by our study protocol. Rare cardiomyopathies, such as arrhythmogenjic right ventricular cardiomyopathy were excluded from analysis, since we a priori decided to focus on non-ischemic cardiomyopathies, which equally affect both ventricles. Future studies are warranted in this context for the detection of subtle LV-dysfunction in patients with biventricular involvement in this disorder. Finally, comparisons of fast-SENC to echocardiographic strain, feature tracking and to native T1-maping techniques were beyond the scope of our study.

## Conclusions

Our study highlights the incremental value of %normal LV and RV myocardium assessed by fast-SENC for the identification of patients with subclinical LV-dysfunction due to non-ischemic cardiomyopathies, compared to conventional CRM imaging variables, such as LVEF and RVEF. Prospective multi-center studies are now warranted to evaluate the ability of fast-SENC to predict cardiac outcomes.

## Supplementary Information

**Additional file 1: Figure S1.** Representative examples of a healthy subject (A) and of a patient with dilated cardiomyopathy (B), hypertrophic cardiomyopathy (C) and cardiac amyloidosis (D). LV and RV strain values are provided.

**Additional file 2: Figure S2.** Associations between %normal LV (A-C) and RV (D-F) myocardium and heart failure stages with different non-ischemic cardiomyopathy groups.

## Data Availability

The data that supports the findings of this study is available from the first and senior author HS and GK upon request.

## Abbreviations

ACC: American College of Cardiology
AHA: American Heart Association
AUC: Area under the curve
bSSFP: Balanced steady state free precession
CAD: Coronary artery disease
CI: Confidence intervals
CMR: Cardiovascular magnetic resonance
CS: Circumferential strain
DCM: Dilated cardiomyopathy
ECG: Electrocardiogram
eGFR: Estimated glomerular filtration rate
fast-SENC: Fast-strain encoded CMR
GCS: Global circumferential strain
GLS: Global longitudinal strain
HCM: Hypertrophic cardiomyopathy
LA: Left atrium
LGE: Late gadolinium enhancement
LS: Longitudinal strain
LV: Left ventricle/left ventricular
LVEF: Left ventricular ejection fraction
MAPSE: Mitral annular plane systolic excursion
NYHA: New York Heart Association
OR: Odds ratio
ROC: Receiver operating characteristics
RV: Right ventricle/right ventricular
RVEF: Right ventricular ejection fraction
TAPSE: Tricuspid annular plane systolic excursion
