# Supplementary figures and images for: Left and right ventricular strain using fast strain-encoded cardiovascular magnetic resonance for the diagnostic classification of patients with chronic non-ischemic heart failure due to dilated, hypertrophic cardiomyopathy or cardiac amyloidosis
Source: J Cardiovasc Magn Reson. 2021 Apr 5;23:45. doi: 10.1186/s12968-021-00711-w (PMC8025329; doi:10.1186/s12968-021-00711-w)

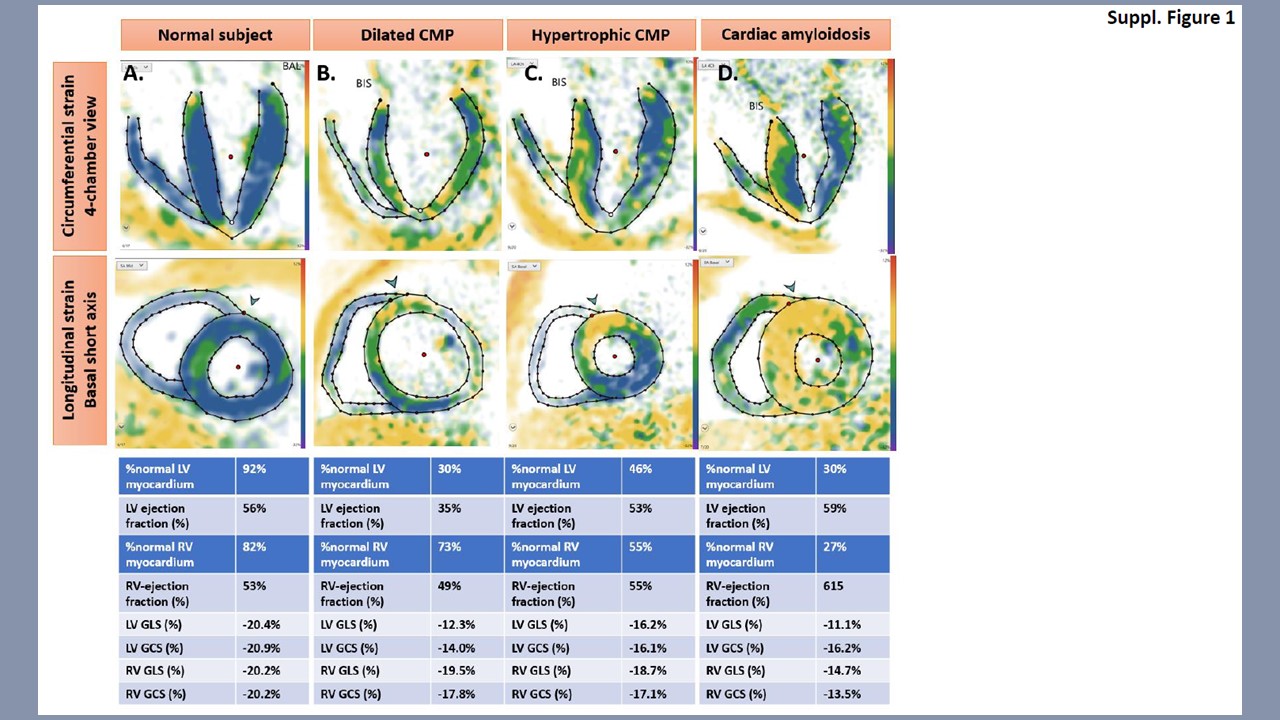

Supplement: Supplementary file 1 — Additional file 1: Figure S1. Representative examples of a healthy subject (A) and of a patient with dilated cardiomyopathy (B), hypertrophic cardiomyopathy (C) and cardiac amyloidosis (D). LV and RV strain values are provided. [file 12968_2021_711_MOESM1_ESM.jpg]

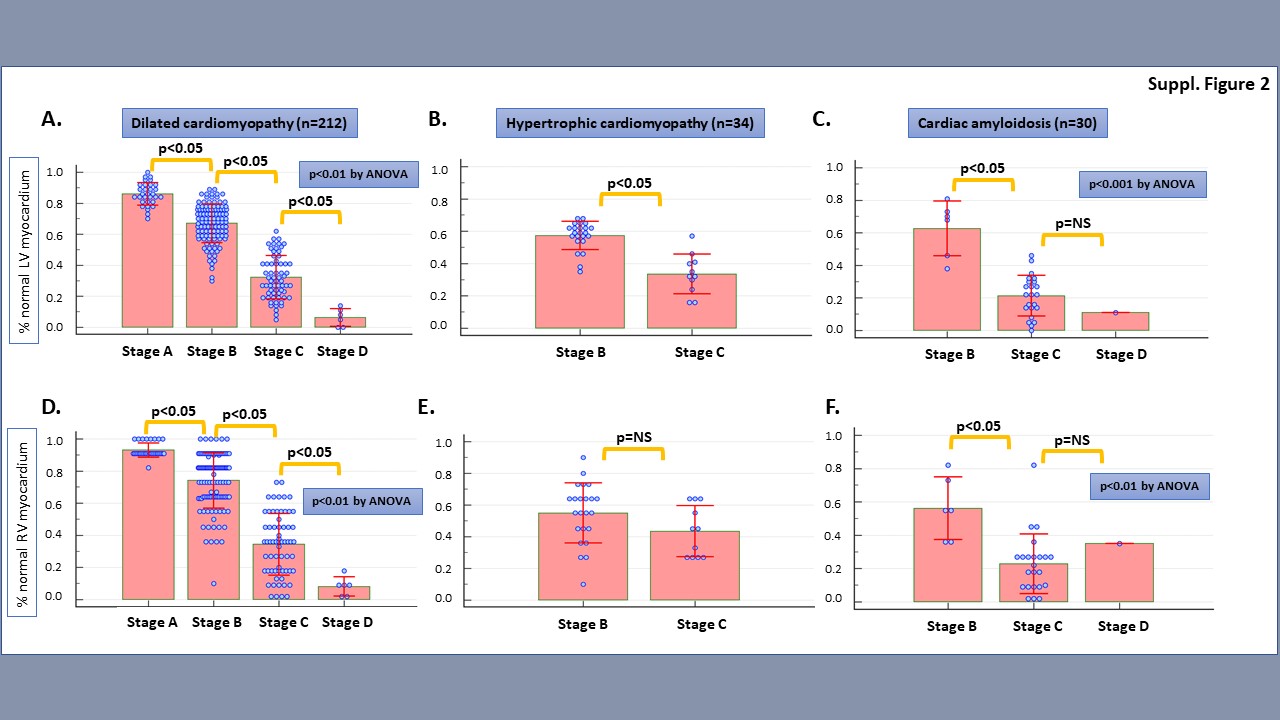

Supplement: Supplementary file 2 — Additional file 2: Figure S2. Associations between %normal LV (A-C) and RV (D-F) myocardium and heart failure stages with different non-ischemic cardiomyopathy groups. [file 12968_2021_711_MOESM2_ESM.jpg]
